# Metal oxide-graphene field-effect transistor: interface trap density extraction model

**DOI:** 10.3762/bjnano.7.128

**Published:** 2016-09-30

**Authors:** Faraz Najam, Kah Cheong Lau, Cheng Siong Lim, Yun Seop Yu, Michael Loong Peng Tan

**Affiliations:** 1 Faculty of Electrical Engineering, Universiti Teknologi Malaysia, 81310 Skudai, Johor, Malaysia; 2 Intel Technology Sdn Bhd, Bayan Lepas Free Industrial Zone, Phase 3, Halaman Kampung Jawa, 11900, Bayan Lepas, Pulau Pinang, 11900, Malaysia; 3 Department of Electrical, Electronic and Control Engineering and IITC, Hankyong National University, Anseong 456-749, Korea

**Keywords:** drain current compact model, interface trap distribution, metal-oxide-graphene field-effect transistor (MOGFET), surface potential

## Abstract

A simple to implement model is presented to extract interface trap density of graphene field effect transistors. The presence of interface trap states detrimentally affects the device drain current–gate voltage relationship *I*_ds_–*V*_gs_. At the moment, there is no analytical method available to extract the interface trap distribution of metal-oxide-graphene field effect transistor (MOGFET) devices. The model presented here extracts the interface trap distribution of MOGFET devices making use of available experimental capacitance–gate voltage *C*_tot_–*V*_gs_ data and a basic set of equations used to define the device physics of MOGFET devices. The model was used to extract the interface trap distribution of 2 experimental devices. Device parameters calculated using the extracted interface trap distribution from the model, including surface potential, interface trap charge and interface trap capacitance compared very well with their respective experimental counterparts. The model enables accurate calculation of the surface potential affected by trap charge. Other models ignore the effect of trap charge and only calculate the ideal surface potential. Such ideal surface potential when used in a surface potential based drain current model will result in an inaccurate prediction of the drain current. Accurate calculation of surface potential that can later be used in drain current model is highlighted as a major advantage of the model.

## Introduction

Graphene has recently attracted a lot of attention. Its 2D nature along with its significantly high carrier mobility (≈15,000 cm^2^/(V·s)) make it an ideal material to replace silicon [1] in the more than Moore era. During deposition of the dielectric layer on graphene as well as from deposition of graphene on the substrate defects may be formed in the film resulting in the presence of trap states; *D*_it_ states (cm^−2^·eV^−1^) at the interface between the dielectric layer and graphene channel [2–3]. These trap states trap mobile carriers degrading the gate field modulation effect, thereby resulting in degraded surface potential.

Popular metal-oxide-graphene field-effect transistor (MOGFET) models do not take into account the detrimental effect of *D*_it_ states on device surface potential [4–5]. Zebrev et al. [6], recently presented a model that takes into account the effect of *D*_it_ states on the device current. A similar approach has been used by [7]. However, Zebrev’s drain current expression is based on the assumption of presence of constant *D*_it_ states over the entire energy range of operation of the device. The assumption does not work generally; recently, significantly varying *D*_it_ distribution has been reported for metal-oxide-graphene (MOG) capacitors [8]. This suggests the need for a model that can analytically calculate the interface trap density of MOGFET devices that could later be used in drain current *I*_ds_ models for efficient *I*_ds_ performance prediction.

This work presents a method to extract interface trap density of MOGFET with the help of device *C*_tot_–*V*_gs_ data. Basic equations and parameters needed to extract interface trap density are explained below. Extraction and verification of extracted trap density is explained following the section below.

## Basic equations and parameters

### Basic equations

**Figure 1:**
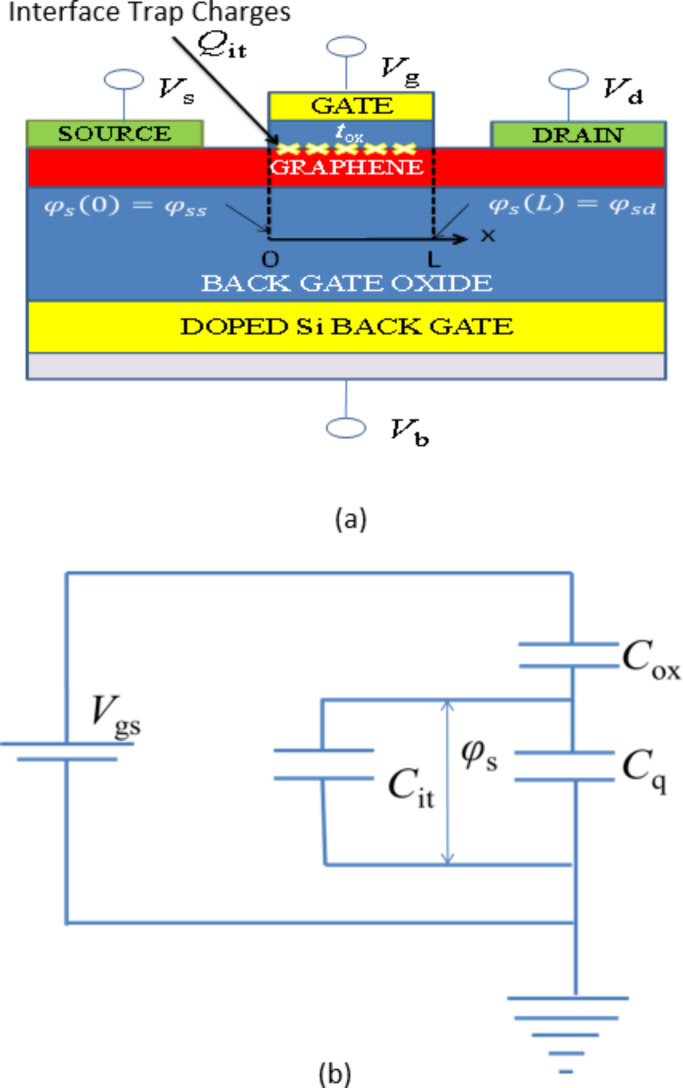
(a) Schematic of MOGFET device. (b) Equivalent capacitive circuit of typical capacitances in MOGFET.

Figure 1a shows the schematic of a typical MOGFET. The channel consists of monolayer graphene with length *L* deposited on a SiO_2_ layer with a p-type doped silicon substrate as the backgate (only top-gated monolayer MOGFET is considered in this work). The gate stack consists of a dielectric layer with thickness *t*_ox_ and a metal gate. *Q*_it_ in Figure 1a refers to the interface trap charge found at the dielectric/channel interface. Figure 1b shows the equivalent capacitive circuit of the typical capacitances in the MOGFET device. In a MOGFET top gate capacitance *C*_ox_ is in series with the parallel combination of interface trap capacitance *C*_it_ which originates from the presence of *D*_it_ states, and *C*_q_ the quantum capacitance.

*C*_q_ is a graphene material property and is given by [9],

[1]
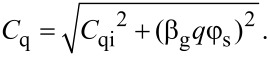


where, *q* is the charge on an electron, φ_s_ is surface potential,


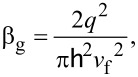



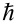
 is the Planck’s constant, *v*_f_ is the fermi velocity (1 × 10^8^ cm^2^/(V·s)), *C*_qi_ is a fitting factor independent of φ_s_, and accounts for the finite *C*_q_ observed at Dirac point (DP) (at which the fermi level *E*_f_ = *q*φ_s_ = 0 = *E*_D_, where *E*_D_ is the energy (eV) at DP).

The total capacitance *C*_tot_ of MOGFET is given by,

[2]
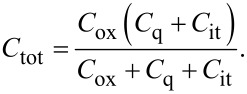


Applying the capacitor divider relation to Figure 1b, the surface potential φ_s_ of MOGFET is given by,

[3]
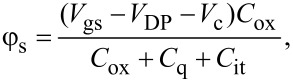


where *V*_gs_ is the gate voltage, *V*_DP_ is the gate voltage at DP known to be caused by the gate-metal/graphene workfunction difference [10], and/or interface trap states [11], and *V*_c_ is the channel voltage drop due to the applied drain bias *V*_ds_ with *V*_c_ = 0 at the source end and *V*_c_ = *V*_ds_ at the drain end.

Solving self-consistently for φ_s_ in Equation 3 and *C*_q_ = (β_g_*q*φ_s_), φ_s_ is given by Equation 4,

[4]
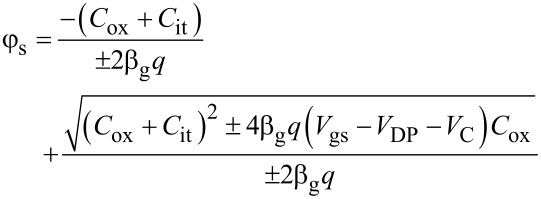


Here, the positive (negative) sign applies when (*V*_gs_ − *V*_DP_ − *V*_c_) *C*_ox_ > 0 (< 0). The sum of *C*_q_ + *C*_it_ in Equation 2 and Equation 3 can be labeled as *C*_x_. The next few paragraphs explain the procedure for extraction of experimental φ_s_, *C*_q_, *C*_it_ and *Q*_it_ parameters of two sample MOGFET devices which are then used in extraction of their *D*_it_ distributions explained in the section “Extraction of interface trap states”.

### Experimental φ_s_, *C*_it_, and *Q*_it_ extraction

Surface potential φ_s_ and *C*_it_ were extracted for two MOGFET devices using experimental *C*_tot_*–V*_gs_ data (from herein referred as *C*_tot_exp_) taken from Device 1 [7], and device 2 [12] (with back-gate bias = 0 V, and *V*_ds_ = 0). The extracted φ_s_ and *C*_it_ parameters obtained using experimental *C*_tot_exp_ data will be referred to as φ_s_exp_ and *C*_it_exp_. The device parameters for both the devices are mentioned in Table 1.

**Table 1:** Device parameters for devices 1 and 2.

Device	Device parameter	MOGFET reported/used value

Device 1 [7]	*C*_ox_ (μF/cm^2^)	1.98
	*V*_DP_ (V)	0.2
	*C*_qi_ (μF/cm^2^)	1
Device 2 [12]	*C*_ox_ (μF/cm^2^)	0.76
	*V*_DP_ (V)	0.11
	*C*_qi_ (μF/cm^2^)	1.6

As mentioned in [12] for Device 2, the DC method used to find *C*_ox_ involves a large amount of ambiguity due to imprecise evaluation of the back-gate capacitance [13], and consequently *C*_ox_. A *C*_ox_ value of 1.00 μF/cm^2^ along with available *C*_q_ and *C*_it_ parameters from [12] in Equation 2 was found to reproduce available *C*_tot_exp_, and *C*_q_ results very well, instead of the reported value of 0.76 μF/cm^2^, the former is used instead in this work. The extraction procedure is described next.

*C*_x_ can be found from Equation 5 which is derived from manipulating Equation 2. Here *C*_tot_ is the respective experimental *C*_tot_–*V*_gs_ data for the two experimental devices and *C*_ox_ is their oxide capacitances mentioned in Table 1.

[5]
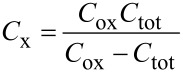


*C*_x_ obtained from the above equation is then substituted in Equation 3 to extract device’s φ_s_ as a function of *V*_gs_, with all the other parameters in Equation 3 known. The extracted φ_s_ is referred to as φ_s_exp_ as device’s surface potential extracted from experimental *C*_tot_–*V*_gs_ data.

Once φ_s_exp_ is obtained, *C*_q_ can be calculated from Equation 1. Finally, device’s *C*_it_ can be obtained using the expression below. The extracted *C*_it_ is referred to as *C*_it_exp_ as device’s interface trap capacitance obtained from experimental *C*_tot_–*V*_gs_ data.

[6]
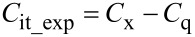


By substituting *C*_it_exp_ in the expression given below, device’s *Q*_it_ can be extracted.

[7]
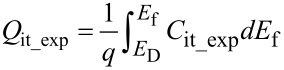


In Equation 7 *E*_f_ = φ_s_exp_. The extracted *Q*_it_ is referred to as *Q*_it_exp_ as the interface trap charge extracted from experimental *C*_tot_–*V*_gs_ data.

The relationship between *C*_it_ and *Q*_it_ is given by

[8]
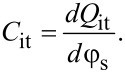


### Extraction of interface trap states

For the extraction, according to standard convention [6] acceptor and donor type traps states were considered for the n-type MOGFET, and p-type MOGFET operation, respectively.

The interface trap charge for both acceptor type and donor type trap states can be calculated from the following [11],

[9]
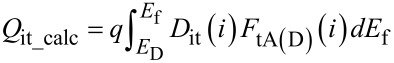


[10]
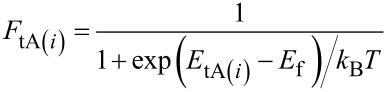


[11]
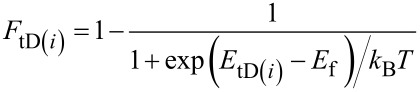


Here, in Equation 9–11, *Q*_it_calc_ denotes the calculated interface trap charge, *F*_tA_ (*F*_tD_) denotes the probability of occupation of *k* acceptor (donor) type trap states, and *E*_tA_ (*E*_tD_) denotes the *i*th energy level of each of these *k* acceptor (donor) type trap state. *D*_it_ is the interface trap density defined at the *i*th energy level. *Q*_it_ can be found by the integral of product of all the *k* trap states with their respective *F*_tA_ (*F*_tD_) between *E*_D_ and *E*_f_.

**Figure 2:**
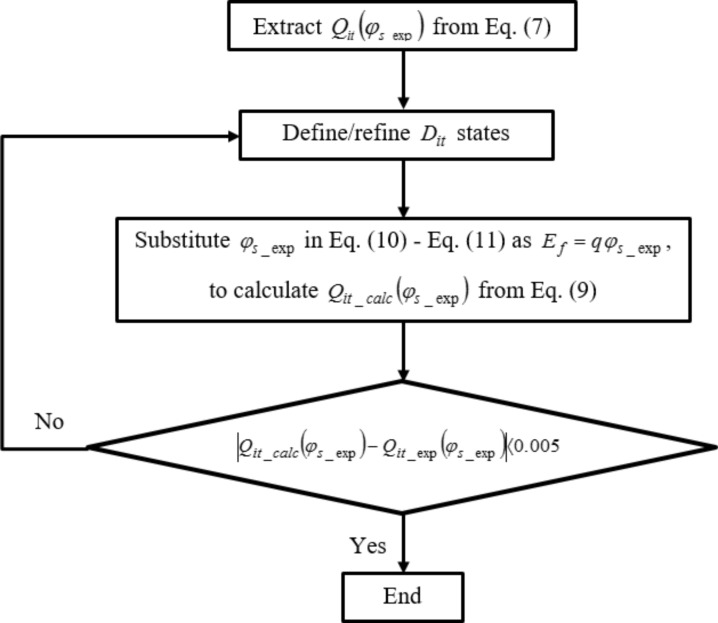
*D*_it_ distribution extraction procedure.

*D*_it_ distribution extraction criteria are based on our earlier work on MoS_2_ MOSFET [14], and are highlighted in Figure 2. The following procedure describes *D*_it_ extraction criteria for MOGFET devices using the two reference experimental devices. As a first step, *Q*_it_exp_ and φ_s_exp_ values are extracted using the procedure outlined in the previous section. Next, the extracted φ_s_exp_ is substituted in Equation 10 and Equation 11 as *E*_f_ = *q*φ_s_exp_ to calculate *F*_tA(D)_ values. These *F*_tA(D)_ values are then used in Equation 9 to find *Q*_it_calc_. In this step and the step prior to this, *D*_it_ values in Equation 9 and *E*_tA(D)_ values in Equation 10 and Equation 11 are fitted for each energy level such that *Q*_it_calc_ obtained using this procedure matches, as a function of φ_s_exp_, experimental *Q*_it_exp_ extracted earlier. This is indicated by step 3 of the flowchart shown in Figure 2.

If *Q*_it_exp_ and *Q*_it_calc_ values as a function of φ_s_exp_ match it means the fitted *D*_it_ values used in Equation 9 to calculate *Q*_it_calc_ were a good fit to reproduce the extracted experimental *Q*_it_exp_. This step enables us to calculate *D*_it_ values.

**Figure 3:**
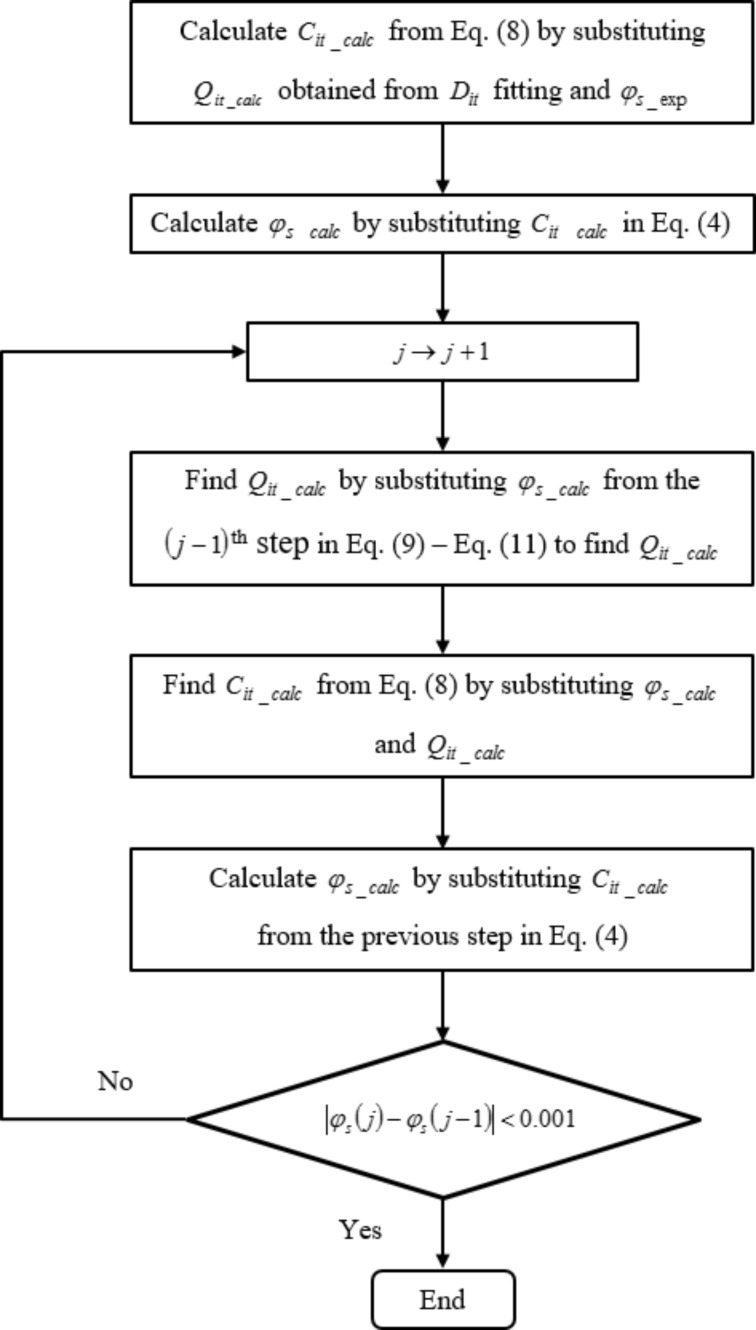
φ_s_calc_/*Q*_it_calc_ self-consistent calculation procedure.

At this point, we have only calculated *Q*_it_calc_ as a function of φ_s_exp_. In order to compare parameters consistently we need to self-consistently find *Q*_it_calc_ as a function of φ_s_calc_, where φ_s_calc_ refers to φ_s_ calculated from Equation 4 using *C*_it_calc_ as the input variable. *C*_it_calc_ refers to *C*_it_ calculated from Equation 8 using *Q*_it_calc_ and φ_s_calc_ as input variables. The self-consistent *C*_it_calc_–φ_s_calc_ calculation procedure is based on our earlier works on MOSFET interface trap drain current modeling [14–15]. The procedure is highlighted in Figure 3 and is described next.

The first step is calculating *C*_it_calc_ from Equation 8 by substituting *Q*_it_calc_ obtained in the previous step (i.e., during the *D*_it_ extraction procedure) and the earlier obtained φ_s_exp_. The calculated *C*_it_ is referred to as *C*_it_calc_. Calcuted *C*_it_calc_ is then substituted in Equation 4 to find φ_s_calc_. This φ_s_calc_ is then substituted back in Equation 9–11 using the already extracted interface trap distribution to calculate *Q*_it_calc_. This *Q*_it_calc_ along with φ_s_calc_ obtained in the previous step is substituted back in Equation 8 to find *C*_it_calc_ which is then substituted in Equation 4 to find φ_s_calc_. This process is repeated back and forth until self-consistency is obtained between *Q*_it_calc_/*C*_it_calc_ and φ_s_calc_. Now we can express *Q*_it_calc_/*C*_it_calc_ as functions of φ_s_calc_, and in turn φ_s_calc_ is calculated using *C*_it_calc_.

Interface trap distribution verification criteria simply implies that

1. *Q*_it_calc_ (as a function of φ_s_calc_) should match well with *Q*_it_exp_ (as a function of φ_s_exp_).
2. *C*_it_calc_ (as a function of φ_s_calc_) should match well with *C*_it_exp_ (as a function of φ_s_exp_).
3. φ_s_calc_ should match well with φ_s_exp_.

**Figure 4:**
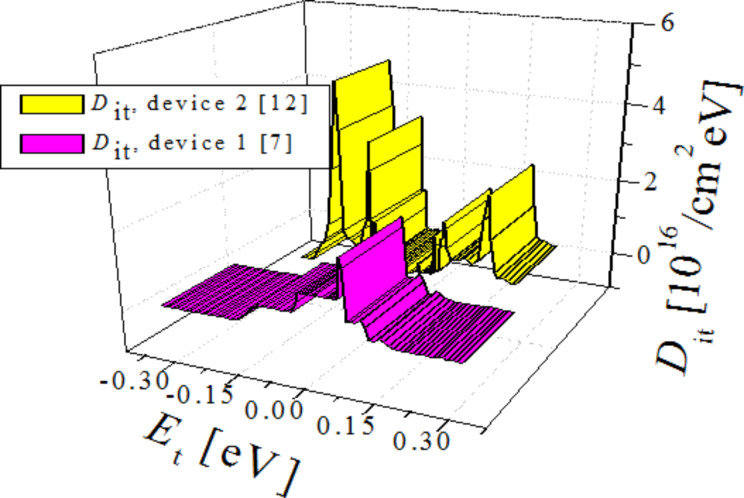
Extracted *D*_it_ distribution, magenta: Device 1, yellow: Device 2.

If the respective calculated and experimental parameters are in reasonable agreement, it proves that the fitted *D*_it_ values used to find the calculated parameters were reasonable (within a tolerance limit) to match well the experimental parameters. The extracted *D*_it_ distribution is shown in Figure 4; magenta for Device 1 and yellow for Device 2.

## Results and Discussion

To prove the validity of the extraction criteria, the extracted experimental parameters, i.e., *Q*_it_exp_, *C*_it_exp_, φ_s_exp_, and *C*_tot_exp_ are compared with the respective calculated, i.e., *Q*_it_calc_, *C*_it_calc_, φ_s_calc_, and *C*_tot_calc_ parameters obtained using the extracted *D*_it_ distribution, as shown in the following.

**Figure 5:**
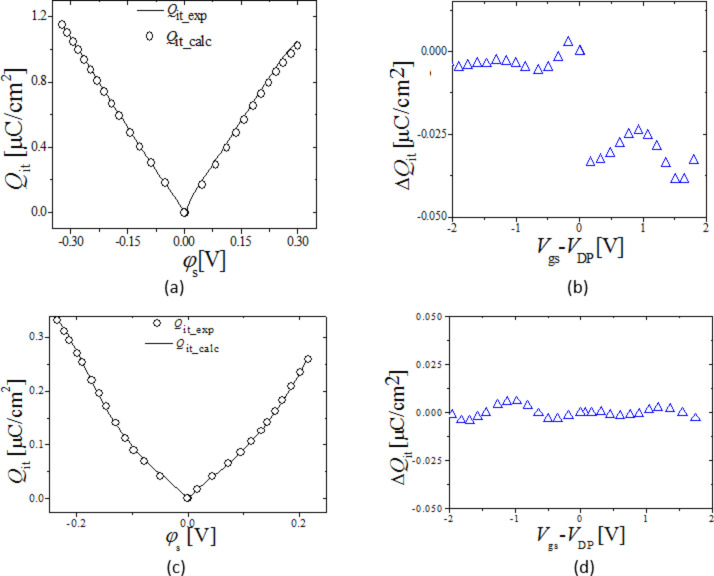
(a) and (c) *Q*_it_ for Device 1 and 2 respectively, symbols: *Q*_it_exp_ from Equation 7 as a function of φ_s_exp_, line: *Q*_it_calc_ calculated from Equation 9–11 as a function of φ_s_calc_. Figure 5b and 5d show the difference in *Q*_it_calc_ and *Q*_it_exp_ as a function of *V*_gs_ for Device 1 and 2 respectively.

Figure 5a and 5b compare for Device 1 and 2, respectively, the extracted *Q*_it_exp_ from Equation 7 (symbols) as a function of φ_s_exp_ with the self-consistently calculated *Q*_it_calc_ as a function of φ_s_calc_. *Q*_it_exp_, and *Q*_it_calc_ are in reasonable agreement as shown by Figure 5b and 5d which show the difference in *Q*_it_calc_ and *Q*_it_exp_ as a function of *V*_gs_, for Device 1 and 2, respectively.

**Figure 6:**
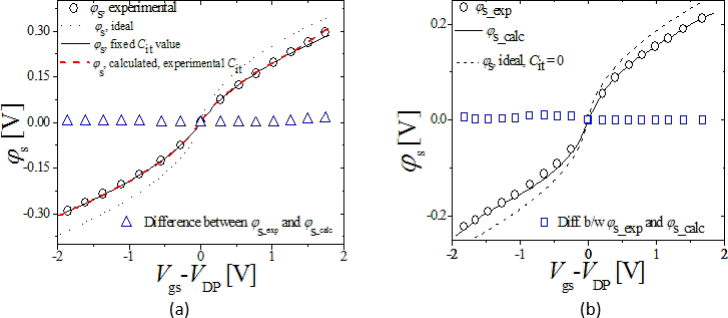
(a) and (b) φ_s_ for Device 1 and 2 respectively as a function of *V*_gs_, symbols: extracted φ_s_exp_, lines: φ_s_calc_ calculated from Equation 4 using the self-consistently obtained *C*_it_calc_/*Q*_it_calc_ from Equation 8–11, dashed lines; φ_s_calc_-ideal from Equation 4 with *C*_it_ = 0. Blue symbols show the difference in φ_s_calc_ and φ_s_exp_.

Figure 6a and 6b show for Device 1 and 2, respectively, the extracted φ_s_exp_ (symbols) as a function of *V*_gs_ − *V*_DP_ compared with φ_s_calc_ (solid line) as a function of *V*_gs_ − *V*_DP_; φ_s_exp_ is in excellent agreement with φ_s_calc_.

Also shown is φ_s_-ideal, calculated from Equation 4 with *C*_it_ = 0 (dashed line). The surface potential calculated with no *C*_it_ = 0 compared with the surface potential calculated considering *C*_it_ clearly indicates that with no *C*_it_ included in the surface potential calculation the result will be an erroneously calculated surface potential. Such an erroneous surface potential if used in surface potential based drain current models will lead to unrealistic prediction of device current. Blue symbols in Figure 6a and 6b show the difference in φ_s_exp_ and φ_s_calc_. As the graph shows, the difference between the two is minimal. The model ensures accurate, realistic calculation of device surface potential by taking into account degradation caused by trap states. This feature could be used to develop more realistic drain current models.

**Figure 7:**
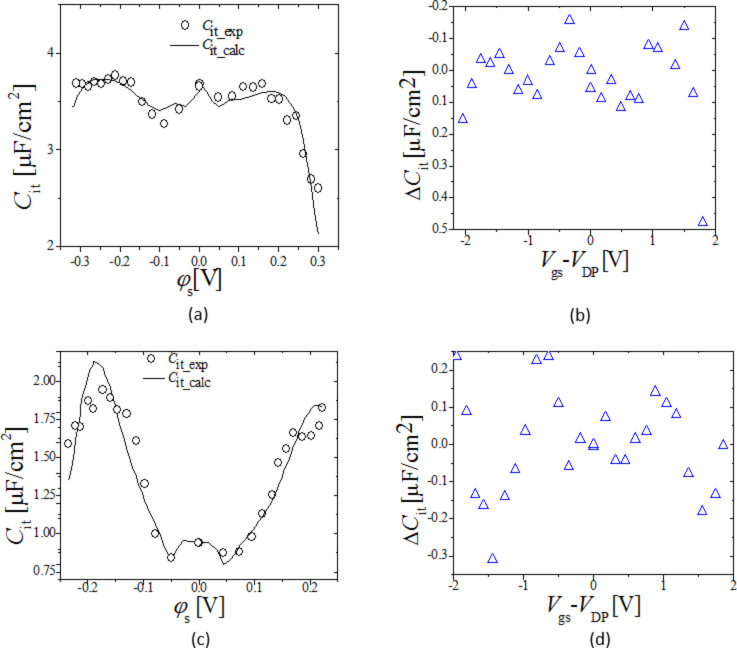
(a) and (c). *C*_it_ for Device 1 and 2 respectively, symbols: *C*_it_exp_ from Equation 6 as a function of φ_s_exp_, line: *C*_it_calc_ calculated from Equation 8–11 as a function of φ_s_calc_. (b) and (d) show the difference between *C*_it_exp_ and *C*_it_calc_ as a function of *V*_gs_ for Device 1, and 2 respectively.

Figure 7a and 7b show for Device 1 and 2 respectively, the extracted *C*_it_exp_ (symbols) from Equation 6, as a function of φ_s_exp_ compared with the *C*_it_calc_ (solid line), as a function of φ_s_calc_; *C*_it_exp_ is in reasonable agreement with *C*_it_calc_. Figure 7b and 7d show difference in *C*_it_exp_ and *C*_it_calc_ as a function of *V*_gs_. The error in *C*_it_calc_ although, higher than *Q*_it_calc_ is still negligible. This is proven when we substitute *C*_it_calc_ in Equation 4 to calculate φ_s_calc_ (when self-consistency is obtained), φ_s_calc_ matches very well with φ_s_exp_ as shown earlier in Figure 6.

**Figure 8:**
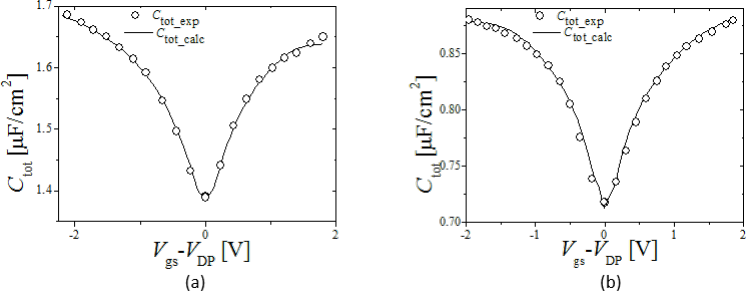
(a) and (b) *C*_tot_ for Device 1 [7] and 2 [12] respectively, symbols: *C*_tot_exp_ as a function of *V*_gs_, lines: *C*_tot_calc_ from Equation 2 as a function of *V*_gs_.

Finally, *C*_tot_exp_ is compared with *C*_tot_calc_ calculated using *C*_q_calc_ from Equation 1, and *C*_it_calc_ obtained above in Equation 2, this is shown in Figure 8a and 8b for Device 1 and 2 respectively; *C*_tot_exp_ (symbols) is in excellent agreement with *C*_tot_calc_ (solid line). All calculated parameters dependent on *D*_it_ states, i.e., *Q*_it_calc_, *C*_it_calc_, φ_s_calc_ and device *C*_tot_calc_ are in excellent agreement with the respective extracted experimental parameters, thereby validating the extracted *D*_it_ distribution.

It must be mentioned part of this work is based on our earlier work on MoS_2_ transistor [14] as briefly mentioned earlier. However, in that work the interface trap density of MoS_2_ transistor was extrated by simply fitting the *Q*_it_ parameter in the device’s drain current (*I*_ds_) model to fit experimental device’s *I*_ds_ with the calculated one from the model. Next, device’s φ_s_ was calculated from the model equation. This φ_s_ was substituted in Equation 9–11 (also used in that work) to fit *E*_tA/D_ and *D*_it_ values to match *Q*_it_ obtained earlier by fitting device’s *I*_ds_. This *D*_it_ distribution extraction procedure is the same in both works. However, in this work, instead of fitting *Q*_it_ in a drain current expression, a thorough analytical framework has been developed, based on fundamental MOGFET device physics, to extract important experimental parameters including *Q*_it_, *C*_it_ and φ_s_ data from experimental *C*_tot_–*V*_gs_ data as highlighted in the section “Experimental φ_s_, *C*_it_, and *Q*_it_ extraction”. Using these experimental parameters as a reference and the framework developed earlier [14–15] an analytical framework was presented to extract the interface trap distribution of MOGFET devices.

To date, to the best of our knowledge this is the only such work in the field. No thorough quantitative, experimental data yet exists on interface trap distribution of graphene transistors. In light of this, this work will be a useful addition to graphene-transistor compact modeling literature.

## Conclusion

In summary, a simple analytic method was introduced to extract the interface trap distribution of MOGFET devices using device’s *C*_tot_–*V*_gs_ data. The model makes use of the basic set of equations used to define device physics of MOGFET devices. Using the procedure mentioned above, interface trap densities of two reference experimental devices were extracted. Device parameters dependent on the extracted interface distribution including the calculated surface potential, interface trap charge, interface trap capacitance and total capacitance matched very well with the respective extracted experimental device parameters. The model enables calculation of device surface potential with the adverse effect of trap charge on device surface potential included. This capability could further be explored in surface potential based MOGFET *I*_ds_ models to help predict MOGFET *I*_ds_–*V*_gs_ performance more accurately by including the effect of interface trap charge on device surface potential.
